# A Novel Homozygous *TTC7A* Missense Mutation Results in Familial Multiple Intestinal Atresia and Combined Immunodeficiency

**DOI:** 10.3389/fimmu.2021.759308

**Published:** 2021-12-15

**Authors:** Wenjun Mou, Shen Yang, Ruolan Guo, Libing Fu, Li Zhang, Weihong Guo, Jingbin Du, Jianxin He, Qinghua Ren, Chanjuan Hao, Jingang Gui, Jinshi Huang

**Affiliations:** ^1^ Laboratory of Tumor Immunology, Beijing Pediatric Research Institute, Beijing Children’s Hospital, Capital Medical University, National Center for Children’s Health, Beijing, China; ^2^ Department of Neonatal Surgery, Beijing Children’s Hospital, Capital Medical University, National Center for Children’s Health, Beijing, China; ^3^ Beijing Key Laboratory for Genetics of Birth Defects, Beijing Pediatric Research Institute; Ministry Of Education (MOE) Key Laboratory of Major Diseases in Children; Genetics and Birth Defects Control Center, Beijing Children’s Hospital, Capital Medical University, National Center for Children’s Health, Beijing, China; ^4^ Department of Pathology, Beijing Children’s Hospital, Capital Medical University, National Center for Children’s Health, Beijing, China; ^5^ Key Laboratory of Advanced Theory and Application in Statistics and Data Science-Ministry Of Education (MOE), School of Statistics, East China Normal University, Shanghai, China; ^6^ Department of Respiratory Medicine, Beijing Children’s Hospital, Capital Medical University, National Center for Children’s Health, Beijing, China; ^7^ Department of Surgical Oncology, Beijing Children’s Hospital, Capital Medical University, National Center for Children’s Health, Beijing, China

**Keywords:** tetratricopeptide repeat domain 7A, multiple intestinal atresia, neonatal sepsis, combined immunodeficiency, prohibited lymphocyte development

## Abstract

Rare autosomal-recessive variants in tetratricopeptide repeat domain 7A (*TTC7A*) gene have been shown to cause intestinal and immune disorders of variable severity. Missense mutations in *TTC7A* gene, usually retaining most of the functional motifs, is associated with relative milder clinical presentations. In this study, we reported a patient who was suffering from severe multiple intestinal atresia (MIA) with combined immunodeficiency (CID) that led to the pyloric diaphragm, ileum atresia, colon stenosis, and multiple episodes of sepsis. In spite of several surgeries and supportive treatment, the patient died of severe sepsis and multiple organ failure at age of 3 months. The whole exome sequencing (WES) of peripheral blood samples identified a novel homozygous *TTC7A* missense mutation (c. 206T>C, p. L69P), inherited from his parents with consanguineous marriage. *In silico* analysis revealed that a hydrogen bond present between Gly65 and Leu69 in the wild-type TTC7A was disrupted by the Leu69Pro mutation. Moreover, this homozygous missense mutation led to a reduced TTC7A expression in lymphocytes and intestinal tissues, accompanied by impeded lymphocyte development. Further studies demonstrated that the PI4K-FAM126A-EFR3A pathway was impaired in colon tissues. Our data strongly support the linkage of severe MIA-CID with the missense mutation in *TTC7A* gene. More knowledge of the TTC7A protein functions will have important therapeutic implications for patients with MIA-CID.

## Introduction

Rare autosomal-recessive variants in tetratricopeptide repeat domain 7A (TTC7A) have been shown to cause intestinal and immune disorders of variable severity (1). The disease phenotype ranges from inflammatory bowel disease (IBD), very-early-onset IBD (VEO-IBD), to multiple intestinal atresia (MIA), accompanied by mild lymphopenia (ELA) or even combined immunodeficiency (CID) (1).

TTC7A consists of nine tetratricopeptide repeat (TPR) domains that mediate multiprotein scaffolding and facilitate the assembly of TTC7A-PI4KIIIα-FAM126A-EFR3 multiprotein complexes (1, 2). Many missense mutations along with the exons of *TTC7A* have been described (1, 2). Complete loss of function due to truncating *TTC7A* mutations would disrupt the TPR domains, thus presented with more severe intestinal and immune phenotypes (ie, MIA-CID) (1). Hypomorphic mutations in *TTC7A* gene, which allow for the residual function, tend to be linked with relative milder clinical presentations such as IBD and less severe immunological involvement like hypogammaglobulinemia (1, 3, 4).

Thus far, majority of the reported missense mutations was hypomorphic with much milder clinical presentations. Here, we described a severe MIA-CID patient with a homozygous missense mutation in *TTC7A* gene not described elsewhere previously. Immunologic assessment illustrated a severe impediment in lymphocyte development. Besides, this mutation led to a reduced TTC7A expression in lymphocytes and intestinal tissues. Further studies demonstrated that the PI4K-FAM126A-EFR3A pathway was impaired in colon tissues. This study adds to the ever-growing knowledge on MIA-CID that might contribute to the better understanding of this challenging disorder.

## Materials and Methods

### Human Sample Collection

This study was approved by the Medical Ethics Committee of Beijing Children’s Hospital and conducted according to Declaration of Helsinki principles. Clinical information (including the demographic information, details of surgery, complications, clinical course, imaging examination results) and blood or tissue samples were obtained from the patient, parents, and controls. Informed consent has been signed by all the participants.

### Preparation of Peripheral Blood Mononuclear Cells (PBMCs)

The PBMCs were freshly isolated from peripheral blood by density gradient centrifugation using Ficoll-Hypaque (GE Healthcare). Briefly, 4 ml freshly isolated EDTA anticoagulated blood was diluted 1:1 with RPMI-1640 medium (Thermo Fisher Scientific), and layered onto 4 ml Ficoll-Hypaque. After centrifuged at 800 g for 20 minutes at room temperature, the buffy layer containing PBMCs was collected, washed and re-suspended in RPMI-1640 medium. Cell viability was determined with trypan-blue (Sigma-Aldrich) staining and counted on an EVE™ automated cell counter (NanoEnTek).

### Histology and Immunohistochemistry

Tissues were fixed in 10% buffered formalin for at least 24 hours, embedded into paraffin. Tissues were then sectioned into 5 μm slice and stained with hematoxylin and eosin (H&E). For Immunohistochemistry, tissue sections were deparaffinized, rehydrated and treated by epitope retrieval using citrate buffer (pH 6.0). The slides were incubated overnight at 4°C with a rabbit anti-human TTC7A antibody (Proteintech, #21600-1-AP) diluted 1:60 in PBS containing 1% BSA. After rinsing three times with PBST, the sections were incubated with biotinylated goat anti-rabbit secondary antibody (Vector Laboratories) and subsequently with streptavidin-labeled HRP (Vector Laboratories). The sections were finally visualized with 3, 3’-diaminobenzidine (DAB) substrate and counterstained with hematoxylin. Digital images were recorded by an Olympus BX53 microscope.

### Whole Exome Sequencing (WES) and Variants Analysis

Genomic DNA was extracted with a QIAamp DNA blood midi kit (Qiagen) using the peripheral blood samples from the patient and parents. SureSelect Human All Exon Kit v6.0 (Agilent Technologies) was used to build an exome library. We performed the targeted regions sequencing with 150 bp pair-end runs by a NovaSeq 6000 system (Illumina). The mean depth of sequencing was > 100×. Raw data were mapped to the GRCh37/hg19 human reference sequence. The *SAMtools* was used to call single nucleotide variants and indels. All the variants were annotated by ANNOVAR. We filtered the common variants with minor allele frequency > 0.05 according to Single Nucleotide Polymorphism databases (gnomAD, dbSNP 147 and 1000 Genomes Project Database) and disease databases (ClinVar and Human Gene Mutation Database, HGMD). Following programs were used to predict the potential impact of the candidate variants on protein function: SIFT (sift.jcvi.org/www/SIFT_enst_submit.html), Polyphen-2 (genetics.bwh.harvard.edu/pph2/), Mutation Taster (www.mutationtaster.org/), and CADD (cadd.gs.washington.edu/). All candidate variants were interpreted according to guidelines of the American College of Medical Genetics and Genomics (ACMG). Sanger sequencing was performed to verify the mutation of *TTC7A* gene. Primers were designed online using Primer 3.0 software. Primers sequence was listed in **Table 1**.

**Table 1:** Primer sequences.

Primers	Sequence (5'-3')
Sanger sequencing	
TTC7A-206mutant-F	AGTCCTGGGAACTCTGTCTT
TTC7A-206mutant-R	TGAAGGCTGACGACTTACCG
qPCR	
18S rRNA-F	GTAACCCGTTGAACCCCATT
18S rRNA-R	CCATCCAATCGGTAGTAGCG
TTC7A-F	GCACCTCAAGGAAGCAGGTT
TTC7A-R	GCTATGCATGATGCGCACG
PI4KA-F	CCATGAAGTGGGCACCTACC
PI4KA-R	CCATACACCCCAAGAGTTGTG
PI4KB-F	CCCCCAGAGCCTGTTCGAC
PI4KB-R	AGTGGGCAGCCAGACTCG
FAM126A-F	GTTGTGGAGGAATGGTTGTCA
FAM126A-R	GACAGGTTCTAGCAACTCACTT
FAM126B-F	CAGAGTAATGGTTGCATTGAAGC
FAM126B-R	AGGGGATAGTGAAAGACAGAACT
EFR3A-F	GGATCGAATTGGTTCTTACCTGG
EFR3A-R	GGCTTAATGCTTTGAGAATGGCA
EFR3B-F	GGTACGTGTGCATTGCTATGG
EFR3B-R	TGGCAAACTTCACAAACGAGT

### Conservation Analysis of Amino Acid Sequences of TTC7A

Amino acid conservation was evaluated using the UGENE database with the sequences of *Homo sapiens, Pan troglodytes, Macaca mulatta, Bos taurus, Mus musculus, Rattus norvegicus, Gallus gallus, Danio rerio* and *Xenopus (Silurana) tropicalis*.

### *In Silico* Analysis of TTC7A

The sequences of TTC7A were obtained from the UniProtKB (UniProtKB: Q9ULT0). The crystal structure of TTC7A (SMTL ID: 5dse.2.A) was used as the template, which was determined by X-ray diffraction at a resolution of 2.9 Å. The structural impact of mutant Leu69Pro was analyzed by PyMOL software. Residue 69 and certain nearby residues within 3 Å were illustrated. For clear demonstration of the inter-residue relationship, some residues were highlighted in colors with the computed hydrogen bonds being labeled.

### Quantification of T-Cell Receptor Excision Circle (TREC) and Kappa-Deleting Recombination Excision Circle (KREC)

DNA extraction was performed from 300 μl blood according to the manufacturer’s instructions (Qiagen). Multiple fluorescence qPCR was used to determine the copy numbers of KREC and TREC, and a real-time PCR assay was performed on ABI 7500 platform (Life Technologies). *RPP30* gene was used as a housekeeping gene. For each PCR reaction, 50 ng of genomic DNA was used in a final volume of 20 μl. The PCR setup was as follows: 1× (30s 95°C), and 45× (5s 95°C, 34s 60°C). All DNA samples were run in triplicates alongside no-template controls. KREC and TREC copy numbers were determined by extrapolating the values from a unique standard curve, which were generated by PCR amplification from serial dilution a known concentration of TREC/KREC plasmids. Primers and probes sequence are available on request.

### Flow Cytometry

Red blood cells-depleted peripheral blood cells were incubated with the fluorochrome-conjugated monoclonal antibodies for 20 minutes at room temperature in the presence of Fc blocking (BD Biosciences) reagents. All the antibodies used were from BD Pharmingen and listed below: CD3 (clone OKT3), CD19 (clone 4G7), CD45RA (clone HI100), CD4 (clone OKT4), CD8 (clone RPA-T8), CD24 (clone ML5), CD38 (clone HIT2), CD16 (clone 3G8), and CD56 (clone B159). The samples were acquired on a BD LSRFortessa flow cytometer (BD Biosciences), and analyzed using FlowJo software (Tree Star).

### Western Blot Analysis

Cell lysates from PBMCs were prepared in cold RIPA lysis buffer supplemented with protease and phosphatase inhibitor cocktails (Roche Life Science). Equivalent amounts of total proteins (20-50 μg) were separated by 10% SDS-PAGE gel electrophoresis and subsequently transferred onto a PVDF membrane (Millipore). After blocking in 5% BSA solution, the membranes were incubated with appropriate primary and secondary antibodies, visualized using ECL substrate. The blots were scanned on Fusion Solo Imaging system (VILBER) and analyzed with ImageJ software. The following primary antibodies were used: TTC7A (Proteintech, 21600-1-AP, 1:300 dilution), β-actin (Cell Signaling, 4970, 1:2000 dilution).

### Quantitative Real-Time PCR (qPCR) Analysis

Total RNA was extracted from a portion of the colectomy specimen using a RNeasy Kit (Qiagen). qPCR was performed using SYBR Green on an ABI 7500 system (Life Technologies). Primer sequences were listed in **Table 1**. The narrow colon tissues of the patient were obtained from surgical resection at the age of 81 days. Control samples were obtained from the noninflammatory location of colon tissues proximal to the dilated segment (relatively normal colon tissue) as controls during the surgery of classic Hirschsprung’s disease (HD). We excluded the possibility of intestinal atresia or CID in both HD patients before the surgery. The selected colon tissues of HD were consistent with the location of the patient in this study.

### RNA Extraction, Sequencing and Analysis

Total RNAs were extracted from PBMCs. The RNA libraries were constructed by poly(A) protocol. Fragmentation was performed using Fragmentation Buffer. The two strands of cDNA were synthesized using a random hexamer primer and M-MuLV Reverse Transcriptase (RNase H Minus) and DNA polymerase I and RNase H, respectively. The Illumina NovaSeq 6000 system was employed to sequence the cDNA libraries with paired-end 150 bp reads.

The paired-end reads were aligned to human reference genome by Hisat v2.2.1 (5) with Ensembl gene annotation (GRCh37/hg19 assembly). The mapped reads were then processed by Stringtie v2.1.4 to quantitate the gene expression against the Ensembl transcript annotation (6). The RNA-seq data of healthy controls were downloaded from National Center for Biotechnology Information (NCBI) Gene Expression Omnibus (GEO) database (GSE111459) (7). The count-based gene expression data was normalized and processed by R DESeq2 package (8). The genes were considered as differentially expressed genes (DEGs) if their fold changes between the patient and healthy controls were greater than 2. The gene set enrichment analysis (GSEA) was conducted to identify the Gene Ontology (GO) terms or pathways overrepresented by the DEGs. The pathways used for GSEA were curated by Kyoto Encyclopedia of Genes and Genomes (KEGG) (9), Reactome (10), and WikiPathway (11), and collected from MSigDB database. The GSEA was implemented by R enricher function in clusterProfiler package (12).

### Statistical Analysis

Differences between groups were calculated using unpaired Student *t*-test with GraphPad Prism 9 software. All experiments were repeated at least three times, and data were presented as the mean ± SD. The statistical significances were indicated as *, *p* < 0. 05; **, *p* < 0.01; ***, *p* < 0.001; ****, *p* < 0.0001.

## Results

### Identification and Intestinal Histopathology of the MIA Patient

**Figure 1:**
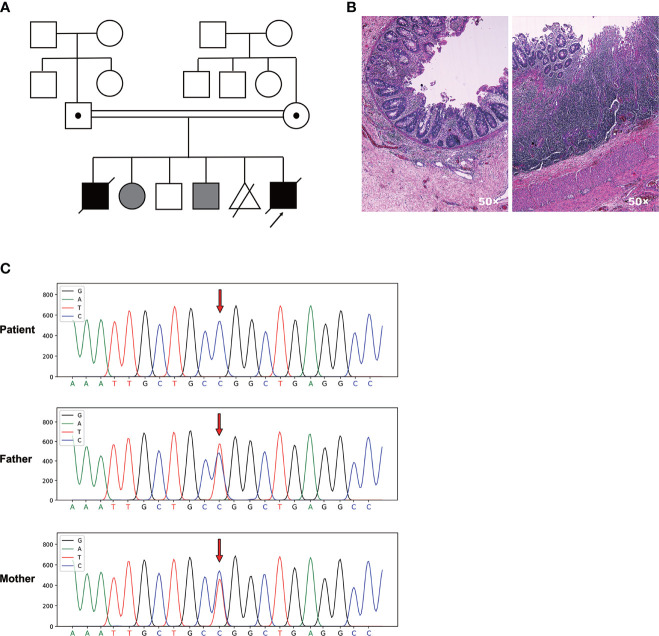
A novel homozygous *TTC7A* missense mutation was identified in the patient with familial MIA. **(A)** Pedigree chart of the proband’s family. Consanguinity is indicated with a double horizontal line. Males are depicted as squares, females as circles, decreased individuals with a diagonal line. Abortion is depicted as a triangle with a diagonal line. Black filled symbols represent individuals who are clinically affected, while grey filled symbols indicate individuals who do not receive medical testing. The carrier is indicated by a dot in squares or circles. The proband who underwent WES is indicated by an arrow. **(B)** H&E staining of the patient’s ileum of the atretic areas shows mucosal erosion, epithelial depolarization, small vascular hyperplasia and dense immune-cell infiltration within the lamina propria. Glands located in the lamina propria are dilated and twisted. **(C)** Sanger sequencing chromatogram depicts the c. 206 T>C missense mutation of *TTC7A*.

The proband was a boy born to consanguineous parents of Chinese origin, and he was the fifth child of the couple (**Figure 1A**). The first child was diagnosed with MIA and died in the first month of life due to postoperative complications including severe septicemia and multiple organ failure. The second and fourth child manifested with constant incidence of vomiting due to digestive obstruction after birth and died in the neonatal period. The third child was an 11-year-old boy without any obvious healthy issues. The mother had a history of induced abortion (the fifth pregnancy, **Figure 1A**). The patient’s uncle (father’s younger brother) is otherwise healthy with congenital absence of the left forearm, and other known relatives did not have congenital malformations or diseases.

The patient was born with a vaginal delivery at an estimated gestational age of 34 weeks. After birth, neonatal intestinal perforation and meconium peritonitis caused by intestinal obstruction were suspected. The patient was diagnosed with MIA involving the pyloric diaphragm, ileum atresia, and colon stenosis after three times of surgery. Furthermore, pathological examination of the ileum adjacent to the atresia areas revealed mucosal erosion, epithelial depolarization, small vascular hyperplasia and dense immune-cell infiltration within the lamina propria. Glands located in the lamina propria were dilated and twisted, with diffused submucosal lymphoproliferative changes (**Figure 1B**).

During hospitalization, the patient experienced seven episodes of sepsis characterized by hyperpyrexia and elevated C-reactive protein (CRP), which in many occasions were accompanied by increased numbers of white blood cell (WBC) and neutrophils (**Supplementary Table 1**). Coinfection of *Klebsiella oxytoca* and *Methicillin-Resistant Staphylococcus epidermidis* were detected by two sets of blood culture drawn from different sites at 21 days after birth. Recurrent and refractory sepsis was diagnosed with poor response to antibiotics including Aztreonam, Amikacin, Meropenem, and Fluconazole. No serious pulmonary infection, vaccine-associated pathogen dissemination, and no signs of fungal and other opportunistic infections had been found. The oftenest episodes of antibiotic-resistant infections could not be explained by the common postoperative complications, which would otherwise be effectively contained by above treatment. Unfortunately, the patient eventually died of respiratory and circulatory failure, electrolyte disturbance, and severe infection at 3 months of age.

### A Novel Homozygous Missense Mutation Was Identified in Exon 2 of *TTC7A* Gene Through WES

To dissect the genetic element that could possibly associate with the disease, we carried out the WES using genomic DNA from the patient and the parents. We identified a novel homozygous missense mutation c. 206T>C in exon 2 of *TTC7A* gene in the patient that had not been documented in the Clinvar and the HGMD databases. Both unaffected father and mother were heterozygous for *TTC7A* missense mutation c. 206T>C. Sanger sequencing confirmed one allele of the *TTC7A* gene was inherited from his father, and the other from the mother (**Figure 1C**). This homozygous missense mutation was predicted as pathogenic by MutationTaster software.

**Figure 2:**
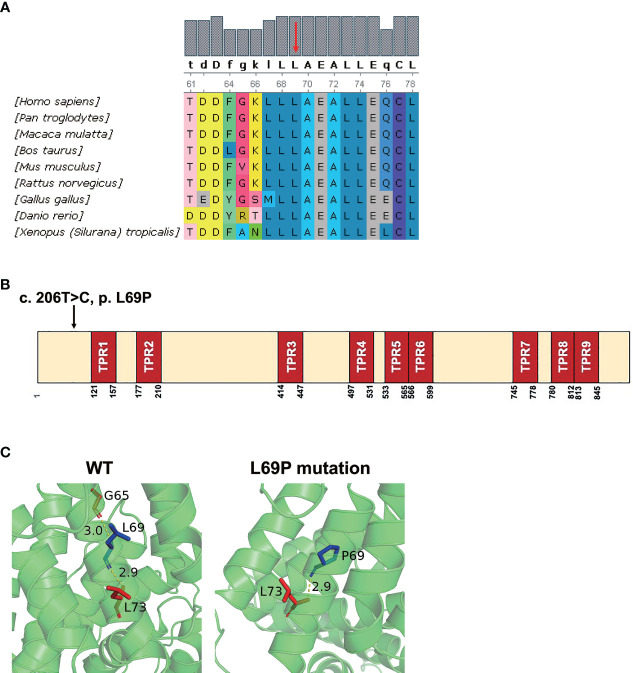
*In silico* analysis of the mutant TTC7A and amino acid conservation of p. L69P. **(A)** Protein alignment indicated that p. L69P amino acids were highly conserved across different species. Residue L69 is shown in red arrow. **(B)** The schematic structure of TTC7A shows the location of the variant identified in this study. Illustration of TTC7A protein with TPR domains in red and the identified mutation is highlighted by an arrow. **(C)** The hydrogen bond presenting between Gly65 and Leu69 in the wild-type TTC7A is disrupted by the L69P mutation. The residue 69 together with certain nearby residues within 3Å is illustrated in the wild-type and the mutant by PyMOL. Computed hydrogen bonds are shown as yellow dashed lines. Residues Leu69/Pro69 are highlighted in blue.

Phylogenetic analysis indicated that the amino acid sequences surrounding the proline 69 residue were highly conserved across different species by UGENE software (**Figure 2A**). As shown in **Figure 2B**, this site-mutation would cause a conversion from leucine 69 into proline in exon 2 (p. Leu69Pro). *In silico* analysis indicated that the hydrogen bond present between Gly65 and Leu69 in the wild-type TTC7A was disrupted by the Leu69Pro mutation (**Figure 2C**).

### The Homozygous *TTC7A* Missense Mutation Led to a T^low^B^-^NK^low^ Immunophenotype

**Figure 3:**
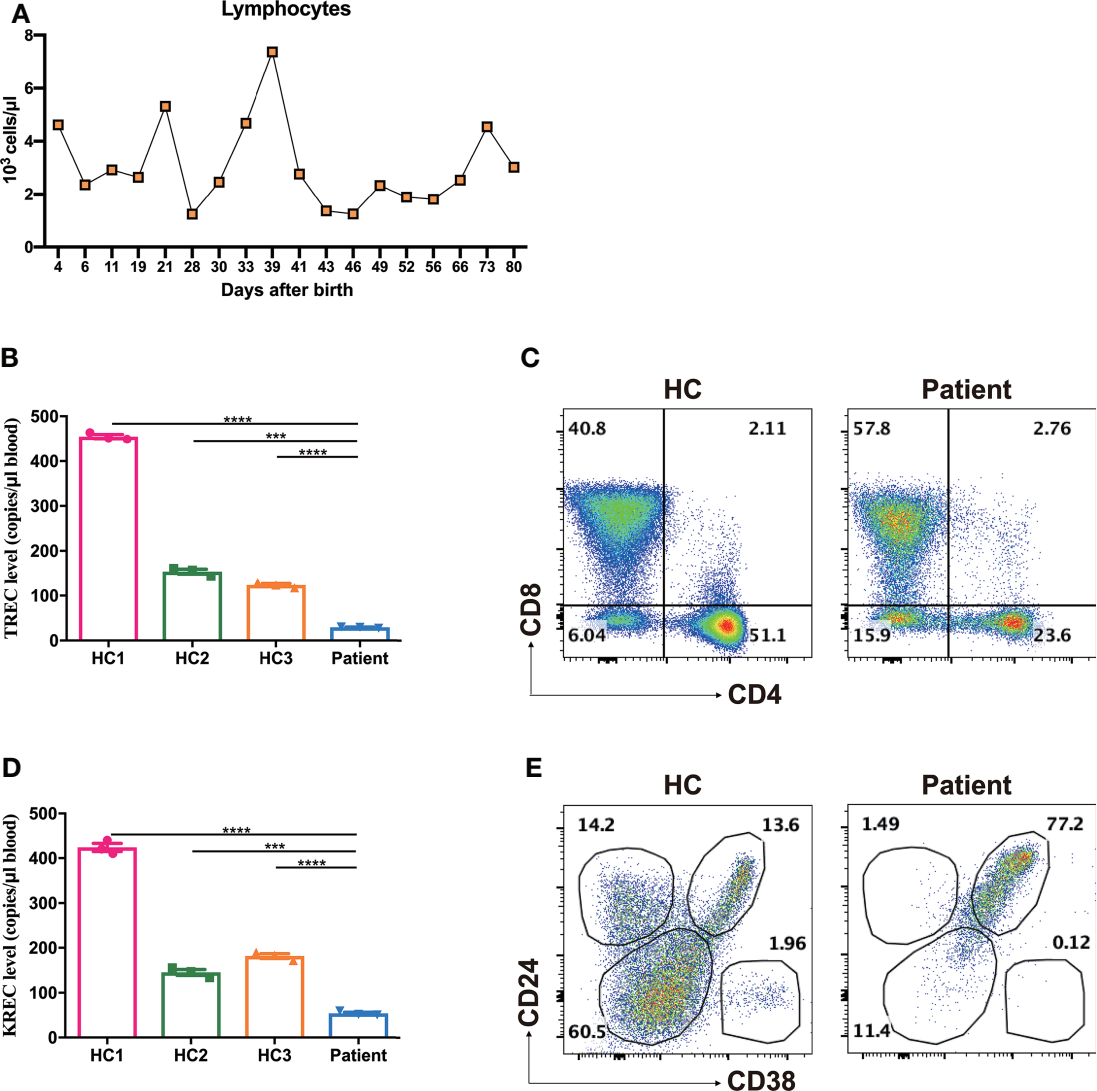
Increased frequencies of DNT, transitional B cells and reduced TREC, KREC levels. **(A)** The absolute lymphocyte counts during treatment. **(B)** Reduced TREC levels of the patient as determined by multiple fluorescence qPCR. ***p < 0.001, ****p < 0.0001. **(C)** Representative dot plots showed markedly elevated frequencies of DNT compared with age- and sex-matched healthy control (HC), as measured by flow cytometry. **(D)** Reduced KREC levels of the patient as determined by multiple fluorescence qPCR. ***p < 0.001, ****p < 0.0001. **(E)** Representative dot plots showed significantly increased frequencies of CD24^bright^CD38^bright^ transitional B cells, as measured by flow cytometry.

A group of MIA patients with *TTC7A* mutation has been shown to be associated with CID (13). In light of multiple episodes of antibiotic-resistant infections, we strongly suspected the immunodeficiency of this patient. The immunologic profiling was delineated by blood cell flow cytometry at the age of 80 days. While the absolute lymphocyte count was kept within the normal range (3020 cells/μl, reference 2400-9500 cells/μl, **Figure 3A**), the frequency of T cells was markedly reduced (6.01% CD3^+^ cells in the patient versus 68.9% and 60.4% in two healthy controls, **Supplementary Figure 1A**). There was a significantly reduced absolute number of T (both CD4 and CD8) cells (CD3, 182 cells/μl, reference 2179-4424 cells/μl; CD4, 47 cells/μl, reference 1461-3018 cells/μl; CD8, 123 cells/μl, reference 556-1687 cells/μl, **Table 2**). In addition, a marked reduction in the proportion and absolute cell number of naive CD4 T-cell (CD4^+^CD45RA^+^) and naive CD8 T-cell (CD8^+^CD45RA^+^) were observed (CD4^+^CD45RA^+^, 6.87%, reference 69.15-88.10%, 13 cells/μl, reference 1170-2595 cells/μl; CD8^+^CD45RA^+^, 16.61%, reference 68.90-94.60%, 31 cells/μl, reference 503-1276 cells/μl, **Table 2**). Moreover, the dropped naive T cells were consistent with a subdued thymic output as the recent thymic emigrants evaluated by our TRECs determination indicated an obvious reduction (29.4 copies/μl in the patient versus 454.8, 153.9, 123.4 copies/μl in three age- and sex-matched healthy controls, **Figure 3B**). Chest X-ray examination also showed that the thymus of the patient was smaller than aged-matched healthy controls. Besides, the patient presented with a markedly elevated double negative T (DNT, CD3^+^CD4^-^CD8^-^, 6.04% versus 15.9%, **Figure 3C**) cells, which suggested that an impeded or skewed T-cell differentiation does exist.

**Table 2:** Immunological phenotypes of the patient.

	Patient		Control value range^a^	
	%	Absolute (cells/μl)	%	Absolute (cells/μl)
**T cells**				
CD3+	6.01	182	54.28-71.67	2179-4424
CD3+CD4+	1.54	47	33.72-52.43	1461-3018
CD3+CD8+	4.06	123	14.08-24.70	556-1687
CD4+CD45RA+	6.87	13	69.15-88.10	1170-2595
CD8+CD45RA+	16.61	31	68.90-94.60	503-1276
**B cells**				
CD19+	0.82	25	17.34-36.03	734-2265
**NK cells**				
CD3-CD56+	5.10	154	5.89-14.85	290-780

a 1–6 months (14).

Likewise, the proportion of CD19^+^ B cells in lymphocytes was markedly reduced (0.82% CD19^+^ cells in the patient versus 18.0% and 22.9% in two healthy controls, **Supplementary Figure 1B**). The patient exhibited a significantly decreased number of peripheral B cells (25 cells/μl, reference 734-2265 cells/μl, **Table 2**). The BCR recombination activity assessed by KREC levels was shown to decrease significantly (54.3 copies/μl in the patient versus 423.8, 145.6, 181.7 copies/μl in three age- and sex-matched healthy controls, **Figure 3D**). The residue B cells in circulation were predominantly transitional B cells (CD24^bright^CD38^bright^) and virtually no CD24^+^CD38^dim^ naive B cells or CD24^+^CD38^-^ memory B cells were presented (naive B cells, 11.4% versus 60.5%; memory B cells, 1.49% versus 14.2%; transitional B cells, 77.2% versus 13.6% **Figure 3E**).

Our study found that the frequency of NK cells in this patient was comparable with that of age- and sex-matched healthy controls (5.10% CD3^-^CD56^+^ cells in the patient versus 5.41% and 5.67% in two healthy controls, **Supplementary Figure 1C**). Whereas, the absolute number of NK cells was significantly reduced (154 cells/μl, reference 290-780 cells/μl, **Table 2**).

Collectively, these results demonstrated that the homozygous *TTC7A* missense mutation led to a compromised lymphocyte development.

### The Homozygous *TTC7A* Missense Mutation Led to Diminished TTC7A Expression

**Figure 4:**
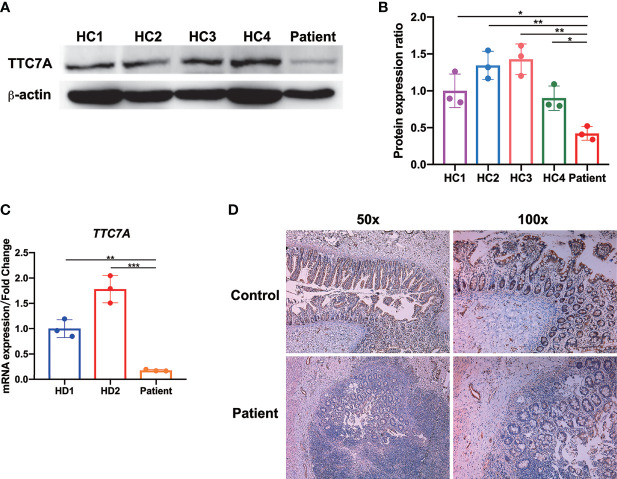
Diminished TTC7A expression in lymphocytes and intestinal tissues. **(A)** Western blot of patient-derived PBMCs showed a severely impairment of TTC7A protein expression compared with age- and sex-matched healthy controls (HC). β-actin was used as an internal loading control in the western blot. The result was a representative of three independent experiments. **(B)** The protein band intensity was quantified and analyzed by ImageJ software *p < 0.05, **p < 0.01. **(C)** The qPCR results demonstrated that patient’s colon tissue had a significant reduction in the amount of *TTC7A* expression compared with those from age- and sex-matched Hirschsprung's disease (HD) patients as control. *TTC7A* expression was normalized to *18S rRNA*, and results were represented as relative expression normalized to the control. **p < 0.01, ***p < 0.001. **(D)** In the patient’s ileum of the atretic areas, immunohistochemistry demonstrated that TTC7A protein expression was diminished compared with the ileum tissue of age- and sex-matched meconium ileus patient (control). Magnification for left panel is ×50, magnification for right panel is ×100.

In the present study, we speculated this novel homozygous missense mutation might affect the TTC7A expression. Indeed, we found that patient PBMCs expressed very low level of TTC7A compared with age- and sex-matched healthy controls by western blot (**Figures 4A, B**). We then tested the *TTC7A* expression in mRNA level using colon tissue of the patient, qPCR analysis indicated that the expression level of *TTC7A* was lower in the patient compared with age- and sex-matched HD patients as control (**Figure 4C**). Immunohistochemical staining of paraffin slice confirmed a reduced level of TTC7A expression in patient ileum tissue compared with that from meconium ileus patient as control (**Figure 4D**). These results confirmed that this homozygous missense mutation in *TTC7A* gene led to a reduced expression in lymphocytes and intestinal tissues.

### The Homozygous *TTC7A* Missense Mutation Led to the Downregulation of Genes Implicated in Lymphocyte Development

**Figure 5:**
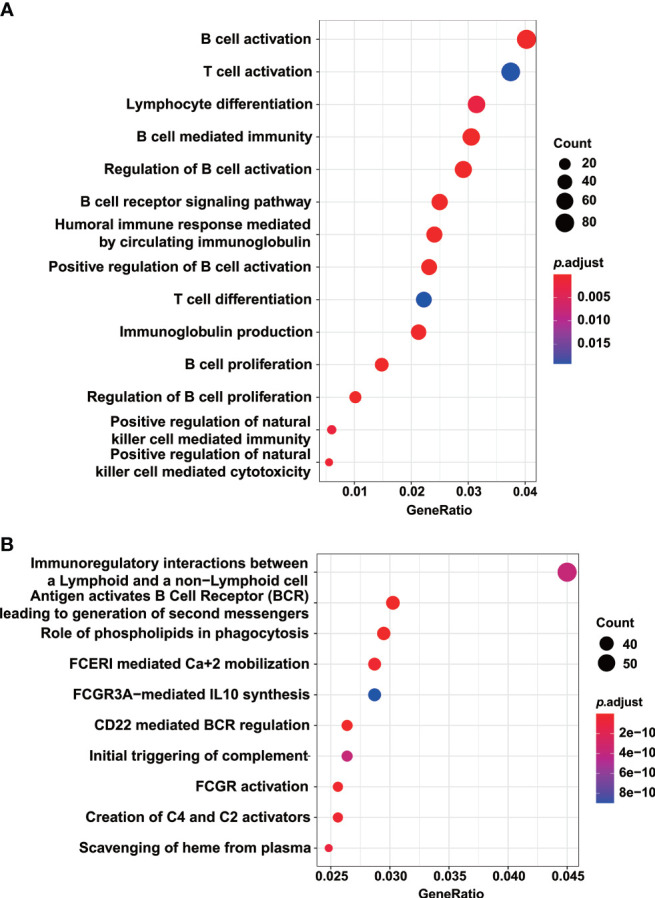
GO and KEGG pathway analysis indicated the downregulation of genes implicated in lymphocyte development. GO terms **(A)** and KEGG pathways **(B)** enriched by the downregulated genes in the patient PBMCs compared with those of healthy controls. Enriched pathways with an adjusted *p* value of less than 0.05 were considered significantly enriched.

To shed light on the functional impact of *TTC7A* variants on the gene expression, we compared the gene expression profile in patient PBMCs with those of healthy controls, and identified a total of 7,396 differentially expressed genes (2,583 upregulated and 4,813 downregulated; adjusted *p*-value < 0.05, fold change > 2). The upregulated and downregulated genes were then subjected to GSEA analysis. The downregulated genes significantly enriched in GO terms and KEGG pathways were clustered into the function of T- and B-cell proliferation, differentiation and activation, as well as NK-cell mediated cytotoxicity (**Figures 5A, B**). The downregulation of genes implicated in lymphocyte functions might explain the T^low^B^-^NK^low^ immune-phenotype we observed in this patient.

### The Homozygous *TTC7A* Missense Mutation Led to in Downregulation of Genes Implicated in the PI4K-FAM126A-EFR3A Pathway of Colon Tissues

**Figure 6:**
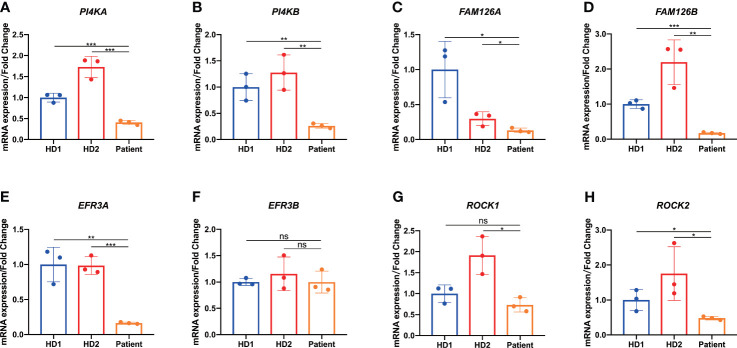
Downregulation of genes implicated in the PI4K-FAM126A-EFR3A pathway of colon tissue. **(A-E, G, H)** qPCR analysis of patient-derived resected narrow colon tissue showed reduced *PI4KA* **(A)**, *PI4KB* **(B)**, *FAM126A* **(C)**, *FAM126B* **(D)**, *EFR3A* **(E)**, *ROCK1* **(G)**, and *ROCK2* **(H)** expression compared with age- and sex-matched patients with Hirschsprung’s disease (HD) as control. **(F)** qPCR analysis of patient-derived resected narrow colon tissue showed comparable *EFR3B* expression compared with HD. *p < 0.05, **p < 0.01, ***p < 0.001, ns, not significant.

It has been confirmed that *TTC7A* mutations directly hinder the transport of PI4KIIIα, a major TTC7A-interacting protein, into plasma membrane for membranous stabilization (15). We next examined the transcription level of *PI4KA* and *PI4KB* in colon tissue and found that *PI4KA* and *PI4KB* expression were significantly reduced in patient colon tissue compared with that of the same tissue location in HD controls (**Figures 6A, B**). It has been shown that the TTC7A-PI4K complex is stabilized by the adaptor protein FAM126 and tethered by EFR3 at the plasma membrane (1, 16). It was our great curiosity to know how these two important factors expressed in patient colon tissue. Through qPCR analysis, we found that patient expressed much lower levels of *FAM126A* and *FAM126B* mRNA compared with that of HD controls (**Figures 6C, D**). TTC7A has been previously described to interact with EFR3 protein, which might serve as a membrane anchor for PI4K (1). Interestingly, we observed a reduced expression of *EFR3A* mRNA in patient colon tissue but not *EFR3B* (**Figures 6E, F**).

Previous studies reported that TTC7A protein could act as a repressor of RhoA signaling pathway, and crucial to normal ROCK function (2, 17). Reduced levels of *ROCK1* and *ROCK2* expression were found in the patient colon tissue as shown in **Figures 6G, H**.

Taken together, these results indicated that the homozygous *TTC7A* missense mutation of the current patient resulted in the downregulation of genes implicated in the PI4K-FAM126A-EFR3A pathway of colon tissues.

## Discussion

*TTC7A* deficiency is an autosomal-recessively inherited disease. Missense mutations were linked with relative milder clinical presentations (4). In this study, we described an infant with a severe MIA-CID caused by homozygous *TTC7A* missense mutation. This missense mutation locates at nucleotide 206 in exon 2 and has not been described before.

The majority of the reported missense mutations were hypomorphic and presented with relative milder clinical phenotypes (1). For example, three patients with E71K homozygous missense mutation lived to teenagers and presented with intermittent IBD episodes (15). These three patients were likely to have missense mutations retaining most of the functional TPR motifs. The patient in current study died after surgeries and supportive treatment, unlike most other patients with *TTC7A* missense mutations. It implied that the 206T>C missense mutation might lead to the disruption of these TPR domains. Previous studies have shown that *TTC7A* mutations that are predicted to result in significant protein truncation and loss of function tend to result in more severe intestinal phenotypes like MIA (15). The diminished TTC7A expression in PBMCs and intestinal tissues of the current patient, as confirmed by western blot, qPCR and immunohistochemical experiments, suggested the loss of TTC7A protein was consistent with the severity of the clinical manifestations.

In *TTC7A*-deficiency patients with MIA, approximately 50% concomitantly present with CID (18, 19). Our patient had severe T and B lymphocytopenia, which might be caused by impeded lymphocyte development. Indeed, the GSEA analysis indicated that the homozygous *TTC7A* missense mutation of the patient led to the downregulation of genes critical for lymphocyte development in PBMCs. Moreover, both TREC and KREC levels of the patient were significantly reduced, and these also suggested the patient had compromised T- and B-cell development.

TRECs are generated during the T-cell development as a result of V(D)J T-cell rearrangements (20). There was evidence that patients with *TTC7A* mutations had low to mild TREC impairment, and MIA patients was found to have extremely low levels of TREC at birth (13, 21). Our patient had low naive T-cell numbers and TREC levels, reflecting the inability of thymus function for producing recent thymic emigrants. Besides, the patient showed markedly elevated DNT cells, and we hypothesized that it might be a result of the impeded or skewed T-cell differentiation.

KRECs are formed in the early stages of development up to mature B cells (22). The KREC numbers in the periphery represent recent bone marrow emigrants. The low KREC levels of the patient were suggestive of reduced B cells or B-cell function. Studies have shown that KREC levels negatively correlated with transitional B cells (23). Patients with p. L346P mutation of *TTC7A* had compromised B-cell development accompanied by increase in the relative levels of transitional B cells (3), which is in line with our finding that majority of the B cells were transitional B cells in the current patient.

Unfortunately, there is no standard treatment for *TTC7A*-deficiency and traditional therapies do little to treat the intestinal disease (MIA and VEO-IBD). Former studies showed that surgical resections for MIA did not prevent the formation of new atresia (24). Intestinal epithelial cell apoptosis and chronic diarrhea are refractory to traditional IBD therapies including immunosuppressive treatments (15, 25). Hematopoietic stem cell transplantation (HSCT) has been reported to restore immunologic defects while the intestinal disease was refractory to HSCT (18, 21). Although many new treatment strategies, including early total enterectomy combined with parenteral nutrition support, immunoglobulin prophylaxis, antibiotics and small-bowel-liver transplant, have been reported successfully, their safety and limitations remain to be evaluated in the future (26, 27). With the understanding of pathobiology of *TTC7A* mutations, the PI4KA-TTC7A-EFR3 or ROCK-TTC7A signaling pathways may lead to the development of effective therapies capable of improving intestinal health (15, 28). Also, early recognition of the immunodeficiency and early intervention with IgG replacement and prophylactic antimicrobials may provide time to make long-term therapeutic decisions for these patients.

In conclusion, *TTC7A* mutations should be investigated in patients with MIA with or without CID. Our study expands the spectrum of *TTC7A* mutations in MIA-CID. Characterization of the role of TTC7A protein in the immune system and intestinal development, as well as in thymic epithelial cells, might have important therapeutic implications in MIA-CID patients in the future.

## Consent to Participate

Clinical information and blood samples were collected from the patient, the parents, and controls, all of whom had given their prior informed consent to participation in the study.

## Data Availability Statement

The data presented in the study are deposited in National Omics Data Encyclopedia (NODE), accession number OEZ007856 (https://www.biosino.org/node/analysis/detail/OEZ007856).

## Ethics Statement

The studies involving human participants were reviewed and approved by Medical Ethics Committee of Beijing Children’s Hospital. Written informed consent to participate in this study was provided by the participants’ legal guardian/next of kin.

## Author Contributions

WM, SY, JG, and JSH designed most of the studies. WM and SY carried out much of the work together with RG, LF, LZ, QR, WG, JD, JXH, and CH. SY, WM, JXH, JG, and JSH analyzed the data. WM wrote up the manuscript with input from SY, JG, JSH, LZ, and RG. All authors contributed to the article and approved the submitted version.

## Funding

This work was supported by grants from National Natural Science Foundation of China (No. 81802491).

## Conflict of Interest

The authors declare that the research was conducted in the absence of any commercial or financial relationships that could be construed as a potential conflict of interest.

## Publisher’s Note

All claims expressed in this article are solely those of the authors and do not necessarily represent those of their affiliated organizations, or those of the publisher, the editors and the reviewers. Any product that may be evaluated in this article, or claim that may be made by its manufacturer, is not guaranteed or endorsed by the publisher.

## Supplementary Material

Supplementary Figure 1 Frequencies of T-cell, B-cell and NK-cell in patient’s blood. Representative dot plots showed markedly reduced frequencies of T-cell **(A)**, B-cell **(B)** compared with age- and sex-matched healthy controls (HC), as measured by flow cytometry. **(C)** Representative dot plots showed that the frequency of NK-cell was comparable with that of HC.
