# Supplementary material for: A Novel Homozygous TTC7A Missense Mutation Results in Familial Multiple Intestinal Atresia and Combined Immunodeficiency
Source: Front Immunol. 2021 Dec 15;12:759308. doi: 10.3389/fimmu.2021.759308 (PMC8714664; doi:10.3389/fimmu.2021.759308)
Supplement: Supplementary file 2 [file Table_1.docx]

**Supplementary Table 1. Clinical characteristics of seven episodes of sepsis**

| **Days after birth (days)** | **Temperature (°C)** | **CRP (mg/L)** | **WBC (×10^9^/L)** | **Neutrophil (×10^9^/L)** | **Neutrophil percentage (%)** |
| --- | --- | --- | --- | --- | --- |
| 4 | 37.9 | 29 | 15.14 | 7.64 | 50.5 |
| 20 | 38.0 | >180 | 8.30 | 5.32 | 64.2 |
| 24 | 37.8 | 68 | 5.89 | 3.29 | 55.9 |
| 30 | 38.0 | 80 | 13.03 | 6.62 | 50.7 |
| 45 | 37.9 | 109 | 6.13 | 4.02 | 59.0 |
| 77 | 37.7 | 79 | 21.82 | 11.15 | 51.2 |
| Reference value | <37.3 | <8 | 4-10 | 0.72-4.60 | 18-46 |
